# Supplementary material for: Steeper size spectra with decreasing phytoplankton biomass indicate strong trophic amplification and future fish declines
Source: Nat Commun. 2024 Jan 9;15:381. doi: 10.1038/s41467-023-44406-5 (PMC10776571; doi:10.1038/s41467-023-44406-5)
Supplement: Supplementary file 1 — Supplementary Information [file 41467_2023_44406_MOESM1_ESM.pdf]

# Steeper size spectra with decreasing phytoplankton indicate strong trophic amplification of future marine biomass decline

# SUPPLEMENTARY INFORMATION

## SUPPLEMENTARY TABLES

**Supplementary Table 1** Summary information on the 41 ecosystems that met our selection criteria for the data compilation. The approximate number of stations or timepoints (6th column) pertains to the number of discrete samplings of the size spectrum, but we did not always have access to the component data, and sometimes only a mean was available. The number of seasons of coverage pertains to the number of seasons of the year (i.e. 1-4). Chl *a* and temperature values presented here are seasonal mean surface values that span these seasons. We have presented both the range of body size used to construct the size spectrum, and an upper limit (very approximate due to the data binning procedure and lack of availability of source data for some studies). These show that some of the studies with the higher ranges of body size included sizes occupied by fish. Studies are ordered by Chl *a* concentration and numbered to correspond to Fig. 1. See main text for numbered superscript references, with additional references listed below.

| Study region | Marine (M) or Lake (FW) | Chl *a* (mg.m$^{-3}$) | Temp (°C) | NBSS slope (SE) | Approx no. of Stns. or timepoints (no. of seasons) | Mass range (o.o.m) Approx maximum Carbon mass | Reference *Unpublished raw size spectra and/or environmental data also supplied by the authors |
|---|---|---|---|---|---|---|---|
| 1.North Pacific Gyre | M | 0.07 | 22.50 | -1.160 (0.0160) | 434 (4) | 7.00 (1 mg) | Rodriguez & Mullin (1986)[1] |
| 2.Lake Limnopolar, Antarctica (outlier – discarded from main analysis) | FW | 0.10 | ~0 | -1.67 | 6 (3) | >7 ( 0.1 mg) | Rochera et al. (2017)[2] |
| 3.NE Aegean Sea Eastern Mediterranean | M | 0.13 | 13.28 | -1.064 (0.0188) | 6 (1) | 9.38 (0.07 mg) | Frangoulis et al (2010, 2017)[3,4]* |
| 4.New England Seamounts, NW Atlantic | M | 0.17 | 24.65 | -1.175 (0.0220) | 1 (1) | 11.92 (1 mg) | Quinones et al. (2003)[5] |
| 5.Cretan Sea, Eastern Mediterranean | M | 0.17 | 20.15 | -1.022 (0.0050) | 43 (4) | 11.80 (2 mg) | unpublished data POSEIDON-E1M3A (Frangoulis & Batziakas)* |
| 6.New England Seamounts, NW Atlantic | M | 0.18 | 24.65 | -1.151 (0.0250) | 1 (1) | 11.92 (1 mg) | Quinones et al. (2003)[5] |
| 7.S Atlantic Gyre | M | 0.21 | 24.00 | -1.080 (0.0123) | 15 (2) | 7.83 (4 mg) | San Martin et al. (2006)[6] |
| 8.New England Seamounts, NW Atlantic | M | 0.25 | 24.65 | -1.145 (0.0200) | 1 (1) | 11.92 (1.3 mg) | Quinones et al. (2003)[5] |
| 9.New England Seamounts, NW Atlantic | M | 0.25 | 24.65 | -1.160 (0.0210) | 1 (1) | 11.92 (1 mg) | Quinones et al. (2003)[5] |
| 10.Sargasso Sea | M | 0.25 | 27.30 | -1.125 (0.0290) | 1 (1) | 11.92 (1 mg) | Quinones et al. (2003)[5] |
| 11.New England Seamounts, NW Atlantic | M | 0.26 | 24.65 | -1.156 (0.0250) | 1 (1) | 11.92 (1 mg) | Quinones et al. (2003)[5] |
| 12.W Tropical Atlantic | M | 0.28 | 27.50 | -1.000 (0.0210) | 13 (2) | 7.83 (4 mg) | San Martin et al. (2006)[5] |
| 13.Sargasso Sea | M | 0.29 | 27.30 | -1.091 (0.0190) | 1 (1) | 11.92 (1 mg) | Quinones et al. (2003 [5] |
| 14.Sargasso Sea | M | 0.33 | 27.30 | -1.141 (0.0250) | 1 (1) | 11.92 (1 mg) | Quinones et al. (2003)[5] |
| 15.Sargasso Sea | M | 0.35 | 27.30 | -1.091 (0.0490) | 1 (1) | 10.58 (0.06 mg) | Quinones et al. (2003)[5] |
| 16.N Atlantic Tropical Gyre | M | 0.38 | 24.70 | -1.060 (0.0202) | 13 (2) | 7.83 (4 mg) | San Martin et al. (2006)[6] |
| 17.Saronic Gulf, near Athens, E Mediterranean | M | 0.40 | 26.00 | -1.160 (0.0136) | 12 (2) | 10.60 (0.1 mg) | Batziakas et al. (2020)[7]* |
| 18.S Subtropical Convergence | M | 0.47 | 17.30 | -1.100 (0.0330) | 9 (2) | 7.83 (4 mg) | San Martin et al. (2006)[6] |
| 19.Eastern Coastal Boundary | M | 0.57 | 24.70 | -0.990 (0.0100) | 2 (2) | 7.83 (4 mg) | San Martin et al. (2006)[6] |
| 20.N Atlantic SubTropical Gyre | M | 0.66 | 22.20 | -1.120 (0.0258) | 10 (2) | 7.83 (4 mg) | San Martin et al. (2006)[6] |

| | | | | | | | |
|---|---|---|---|---|---|---|---|
| 21.Lake Michigan, USA | FW | 0.70 | 14.20 | -1.040 (0.0493) | 36 (2) | 15.95 (4 mg) | Sprules & Goyke (1994)[8]* |
| 22.Lake Superior Canada/USA, 1970s | FW | 0.82 | 9.60 | -1.100 (0.0152) | 11 (3) | 9.80 (1 mg) | Sprules & Munawar (1986)[9]* |
| 23.Lake Superior, Canada/USA, 2000s | FW | 0.90 | 9.60 | -1.068 (0.0060) | 147 (2) | 10.80 (100 mg) | Yurista et al. (2014)[10] Chapra & Dolan (2012)[11] |
| 24.SW Atlantic Shelves | M | 0.90 | 8.00 | -1.130 (0.0569) | 3 (2) | 7.83 (4 mg) | San Martin et al. (2006)[6] |
| 25.Western English Channel | M | 1.06 | 12.49 | -1.106 (0.0044) | 275 (4) | 10.50 (0.3 mg) | Atkinson et al (2021)[12]* |
| 26.N Atlantic Drift | M | 1.10 | 17.00 | -1.13 (0.0357) | 8 (2) | 7.83 (4 mg) | San Martin et al. (2006)[6] |
| 27.Lake Malawi, Malawi, Africa | FW | 1.40 | 26.50 | -1.040 (0.0300) | 500 (4) | 14.10 (140 g) | Sprules (2008)[13]* |
| 28.Scotia Sea, Southern Ocean | M | 1.44 | 2.00 | -1.075 (0.0075) | 28 (3) | 12.50 (30 g) | Tarling et al. (2012)[14] |
| 29.Lake Huron, Canada/USA | FW | 2.10 | 10.30 | -1.020 (0.0346) | 8 (3) | 8.47 (0.04 mg) | Sprules & Munawar (1986)[9]* |
| 30. 25 Oligotrophic inland lakes in Ontario, Canada | FW | 2.30 | 18.60 | -0.980 (0.0080) | 225 (3) | 9.73 (0.8 mg) | Sprules et al. (1983)[15]* |
| 31.Lough Maumwee, Ireland | FW | 2.44 | 17.30 | -1.074 (0.0780) | 3 (2) | 9.33 (0.02 mg) | de Eyto & Irvine (2007)[16]* |
| 32.Lake Ontario, Canada/USA | FW | 2.60 | 14.99 | -1.020 (0.0418) | 87 (3) | 16.85 (3700 g) | Sprules & Goyke (1994)[8]* |
| 33.Lake Constance | FW | 3.81 | 9.68 | -1.034 (0.0035) | 377 (4) | 10.54 (0.09 mg) | Gaedke (1992)[17]* |
| 34.Lake St Clair Canada/USA | FW | 3.86 | 19.60 | -0.900 (0.0372) | 8 (3) | 7.67 (0.04 mg) | Sprules & Munawar (1986)[9] |
| 35.Lough Carra, Ireland | FW | 4.19 | 16.60 | -1.008 (0.1305) | 3 (2) | 9.33 (0.02 mg) | de Eyto & Irvine (2007) [16]* |
| 36.Lough Gara Ireland | FW | 9.42 | 16.50 | -1.089 (0.0561) | 3 (2) | 9.33 (0.02 mg) | de Eyto & Irvine (2007) [16]* |
| 37.Lough Gur, Ireland | FW | 14.59 | 17.50 | -0.958 (0.0356) | 3 (2) | 9.63 (0.03 mg) | de Eyto & Irvine (2007)[16]* |
| 38.Lough Mullagh, Ireland | FW | 28.45 | 15.20 | -0.964 (0.1913) | 3 (2) | 9.63 (0.03 mg) | de Eyto & Irvine (2007)[16]* |
| 39.Lough Ramor, Ireland | FW | 30.28 | 16.96 | -1.000 (0.0622) | 3 (2) | 9.93 (0.07 mg) | de Eyto & Irvine (2007) [16]* |
| 40.Muggelsee, Germany | FW | 39.30 | 12.50 | -1.036 (0.0073) | 70 (4) | 9.93 (0.05 mg) | Gaedke et al. (2004)[18]* |
| 41.Arendsee, Germany | FW | 41.97 | 11.00 | -0.941 (0.0300) | 48 (4) | 9.63 (0.01 mg) | Tittel (1998)[19] |

**Supplementary Table 2.** General Linear Models (GLM) examining the effects of covariates $Log_{10}$[Chl *a*] (mg Chl *a* $m^{-3}$), denoted *C*, and Temperature(°C), denoted *T*, on the slope of the Normalised Biomass Size Spectrum, *N*. $N_w$ denotes the slope values that have been weighted according to the degree of seasonal sampling coverage (see Methods). Significant *P* values ($P < 0.05$) are in bold. We selected our "best" model (i.e. equation in bold) based on the fact that: first, $Log_{10}$[Chl a] was the only covariate that was consistently and significantly related to *N* across all model combinations. Second, the LOWESS fit to the data (**Supplementary Fig. 1d**) suggested a non-linear relationship between *N* and $Log_{10}$[Chl a] as also suggested by previous studies[9,12,20]. Third, unweighted data provided very similar results to a series of data weightings and data exclusions (**Supplementary Fig. 1a**) so we chose the simplest option, namely to use unweighted data.

| Analysis of Variance | | | | | | $R^2$ % (adj) | Regression Equation |
|---|---|---|---|---|---|---|---|
| Source | DF | Adj SS | Adj MS | F-value | P-value | | |
| *C* | 1 | 0.08109 | 0.08109 | 29.3 | **<0.001** | 42.1 | $N = -1.06849 + 0.0624\,C$ |
| Error | 38 | 0.10519 | 0.00277 | | | | |
| *C* | 1 | 0.05772 | 0.05772 | 20.4 | **<0.001** | **40.8** | $\mathbf{N = -1.0651 + 0.0663C - 0.0065\,C^2}$ |
| $C^2$ | 1 | 0.00052 | 0.00052 | 0.18 | 0.670 | | |
| Error | 37 | 0.10466 | 0.00283 | | | | |
| *C* | 1 | 0.04692 | 0.04692 | 16.26 | **<0.001** | 39.6 | $N = -1.0790 + 0.0711C - 0.0085C^2 + 0.00079T$ |
| $C^2$ | 1 | 0.00083 | 0.00083 | 0.29 | 0.594 | | |
| *T* | 1 | 0.00074 | 0.00074 | 0.26 | 0.615 | | |
| Error | 36 | 0.10381 | 0.00289 | | | | |
| *T* | 1 | 0.01648 | 0.01648 | 3.69 | 0.062 | 6.5 | $N = -1.0097 - 0.00308T$ |
| Error | 38 | 0.17000 | 0.00447 | | | | |
| *T* | 1 | 0.00905 | 0.00905 | 2.16 | 0.150 | 12.4 | $N = -1.1202 + 0.01178T - 0.000426T^2$ |
| $T^2$ | 1 | 0.01496 | 0.01496 | 3.57 | 0.067 | | |
| Error | 37 | 0.15484 | 0.00419 | | | | |
| *T* | 1 | 0.00151 | 0.00151 | 0.52 | 0.474 | 39.8 | $N = -1.1097 + 0.00495T - 0.000131T^2 + 0.0618C$ |
| $T^2$ | 1 | 0.00123 | 0.00123 | 0.43 | 0.517 | | |
| *C* | 1 | 0.05132 | 0.05132 | 17.85 | **<0.001** | | |
| Error | 36 | 0.10352 | 0.00288 | | | | |
| *C* | 1 | 0.06504 | 0.06504 | 22.97 | **<0.001** | 40.7 | $N = -1.0794 + 0.00058T + 0.0651C$ |
| *T* | 1 | 0.00043 | 0.00043 | 0.15 | 0.70 | | |
| Error | 37 | 0.10475 | 0.00283 | | | | |
| *C* | 1 | 0.04107 | 0.04107 | 14.03 | **<0.001** | 38.7 | $N = -1.1121 + 0.00560T - 0.000143T^2 + 0.0682C - 0.0096C^2$ |
| $C^2$ | 1 | 0.00107 | 0.00107 | 0.36 | 0.550 | | |
| *T* | 1 | 0.00189 | 0.00189 | 0.64 | 0.428 | | |
| $T^2$ | 1 | 0.00147 | 0.00147 | 0.50 | 0.484 | | |
| Error | 35 | 0.10245 | 0.00293 | | | | |
| *C* | 1 | 0.16400 | 0.16400 | 25.41 | **<0.001** | 38.5 | $N_w = -1.06257 + 0.0565C$ |
| Error | 38 | 0.24523 | 0.24523 | | | | |
| *C* | 1 | 0.01197 | 0.01197 | 18.18 | **<0.001** | 37.3 | $N_w = -1.0593 + 0.0609C - 0.0067C^2$ |
| $C^2$ | 1 | 0.00166 | 0.00166 | 0.25 | 0.619 | | |
| Error | 37 | 0.24357 | 0.00658 | | | | |
| *C* | 1 | 0.12054 | 0.12054 | 18.54 | **<0.001** | 38.0 | $N_w = -1.0905 + 0.0711C - 0.0105C^2 + 0.00185T$ |
| $C^2$ | 1 | 0.00387 | 0.00387 | 0.6 | 0.445 | | |
| *T* | 1 | 0.00952 | 0.00952 | 1.46 | 0.234 | | |
| Error | 36 | 0.23406 | 0.00650 | | | | |
| *T* | 1 | 0.01455 | 0.01455 | 1.4 | 0.244 | 1.02 | $N_w = -1.0205-0.00195T$ |
| Error | 38 | 0.39465 | 0.01039 | | | | |
| *T* | 1 | 0.02060 | 0.02060 | 2.09 | 0.157 | 6.01 | $N_w = -1.1131 + 0.01109T - 0.000388T^2$ |
| $T^2$ | 1 | 0.02976 | 0.02976 | 3.02 | 0.091 | | |
| Error | 37 | 0.36490 | 0.00986 | | | | |
| *T* | 1 | 0.00415 | 0.00415 | 0.63 | 0.431 | 37.6 | $N_w = -1.1141 + 0.00509T - 0.000109\,T^2 + 0.0606C$ |
| $T^2$ | 1 | 0.00210 | 0.00210 | 0.32 | 0.575 | | |
| *C* | 1 | 0.12907 | 0.12907 | 19.70 | **<0.001** | | |
| Error | 36 | 0.23583 | 0.00655 | | | | |

|  |  |  |  |  |  |  |  |
|---|---|---|---|---|---|---|---|
| *C*<br>*T*<br>Error | 1<br>1<br>37 | 0.15673<br>0.00730<br>0.23793 | 0.15673<br>0.00730<br>0.00661 | 24.37<br>1.14 | **<0.001**<br>0.294 | 38.7 | $N_w$ = -1.0908 + 0.0631*C* + 0.00157*T* |
| *C*<br>$C^2$<br>*T*<br>$T^2$<br>Error | 1<br>1<br>1<br>1<br>35 | 0.10900<br>0.00460<br>0.00559<br>0.00283<br>0.23122 | 0.10900<br>0.00460<br>0.00559<br>0.00283<br>0.00661 | 16.50<br>0.70<br>0.85<br>0.43 | **<0.001**<br>0.410<br>0.364<br>0.517 | 37.04 | $N_w$ = -1.1177 + 0.0690*C* - 0.0116$C^2$ + 0.0056*T* - 0.000129$T^2$ |

**Supplementary Table 3.** Results of a bootstrap analysis of the 8 unweighted models (i.e. the first eight models in **Supplementary Table 2**). The bold model 2 is the one we selected and is illustrated in **Fig. 2 a**. These results pertain to 10,000 bootstraps of the resampled NBSS slope values (see Methods). This procedure allows for error propagation, based on the uncertainty in each determination of the NBSS slope, and consequently model *P*- values are substantially greater than those in **Supplementary Table 2**. However, in both analyses the main finding is that Chl *a* is a much better predictor of NBSS slopes than is water temperature.

| Model No. | Model parameters; Mean (Standard Error) |  |  |  |  | Model Summary statistics |  |  |
|---|---|---|---|---|---|---|---|---|
|  | Intercept | C | $C^2$ | T | $T^2$ | $R^2$, %; Median (IQR) | F-statistic; Median (IQR) | p-value; from median F-stat |
| 1 | -1.0685*** (0.0074) | 0.0628*** (0.0155) | X | X | X | 30.6 (14.4) | 18.2 (11.5) | 0.0001 |
| **2** | **-1.0651*** (0.0094)** | **0.0663*** (0.0121)** | **-0.0065 (0.0153)** | **X** | **X** | **29.6 (14.3)** | **9.2 (5.5)** | **0.0006** |
| 3 | -1.0788*** (0.0166) | 0.0712*** (0.0148) | -0.0087 (0.0149) | 0.0008 (0.0009) | X | 28.2 (14.7) | 6.1 (3.7) | 0.0018 |
| 4 | -1.1120*** (0.0278) | 0.0685*** (0.0134) | -0.0098 (0.0155) | 0.0056 (0.0041) | -0.0001 (0.0001) | 27.0 (15.4) | 4.6 (2.8) | 0.0043 |
| 5 | -1.0097*** (0.0191) | X | X | -0.0031*** (0.0007) | X | 3.7 (3.9) | 2.5 (1.7) | 0.1217 |
| 6 | -1.1199*** (0.0290) | X | X | 0.0116*** (0.0049) | -0.0004*** (0.0001) | 7.4 (7.2) | 2.6 (1.6) | 0.0910 |
| 7 | -1.0797*** (0.01634) | 0.0653*** (0.0182) | X | 0.0006 (0.0009) | X | 29.1 (15.3) | 9.0 (5.8) | 0.0007 |
| 8 | -1.1100*** (0.02818) | 0.0620*** (0.0176) | X | 0.0050 (0.0040) | -0.0001 (0.0001) | 27.9 (16.1) | 6.0 (4.0) | 0.0019 |

*** $P < 0.001$
** $P < 0.01$
* $P < 0.05$

# SUPPLEMENTARY FIGURES

**Supplementary Figure 1. The strong relationships of NBSS slope with Chl *a* and lack of statistical relationship with temperature are robust to data selection and weighting**

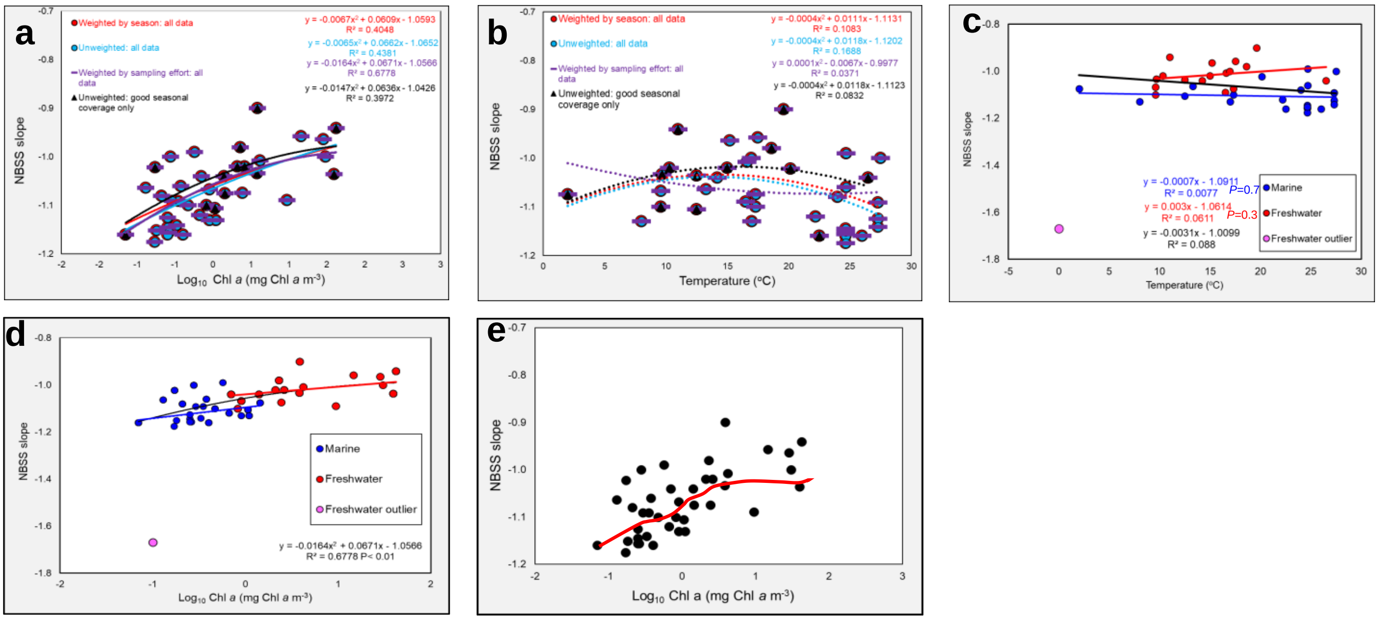


Using General Linear Models and regressions of the data sets, we tested how robust our conclusions were to outliers, differing combinations of data and differing weighting of points based on sampling effort. **a** Relationships between Normalised Biomass Size Spectrum (NBSS) slope and Log Chl *a* based on 40 ecosystems with: weighting according to number of seasons of the year sampled (weighted from 1 to 4); no weighting; weighted according to sampling effort (i.e. number of samplings listed in **Supplementary Table 1**); and unweighted data points but based only on those sampled during at least 3 seasons of the year. All of these relationships were highly significant ($P < 0.001$) **b** Equivalent plots for the NBSS-temperature relationship: none of these relationships was significant ($P > 0.05$). **c** Relationships of NBSS with temperature, with points coded according to whether they are marine (blue) or freshwater (red). Separate regression lines and regression statistics are presented for the marine, freshwater and all data except the pink outlier (black line and regression). These support our previous analyses, showing no evidence for temperature relationships within these subsets of the data.

The pink point represents an outlier station (ecosystem no. 2 in **Fig.1** and **Supplementary Table 1**: Lake Limnopolar with highly negative NBSS slope, thought by the authors to be due to incomplete sampling of predators lurking near the bottom of the lake. **d** The NBSS- Log Chl *a* relationships with points coded according to whether they are marine or freshwater. The separate linear regressions fitted to marine and freshwater subsets of the data, show that the positive relationship between NBSS slope and Log (Chl *a*) is preserved for both subsets of data. For reference, the polynomial relationship is shown for all data (black line). Inclusion of the pink outlier maintains a significant relationship but has a large effect on the slope at low Chl *a* concentrations, so it was removed. **e** Same data as in panel a except for the outlier, with LOWESS line fitted. This shows a flatter relationship between NBSS slope and Log Chl *a* at high Chl *a* concentrations, as found previously[9,20].

**<u>Supplementary Figure 2.</u> Case study analysis of three ecosystems of increasing nutrient status: highly varied seasonal relationships between bloom dynamics, temperature and NBSS slope.**

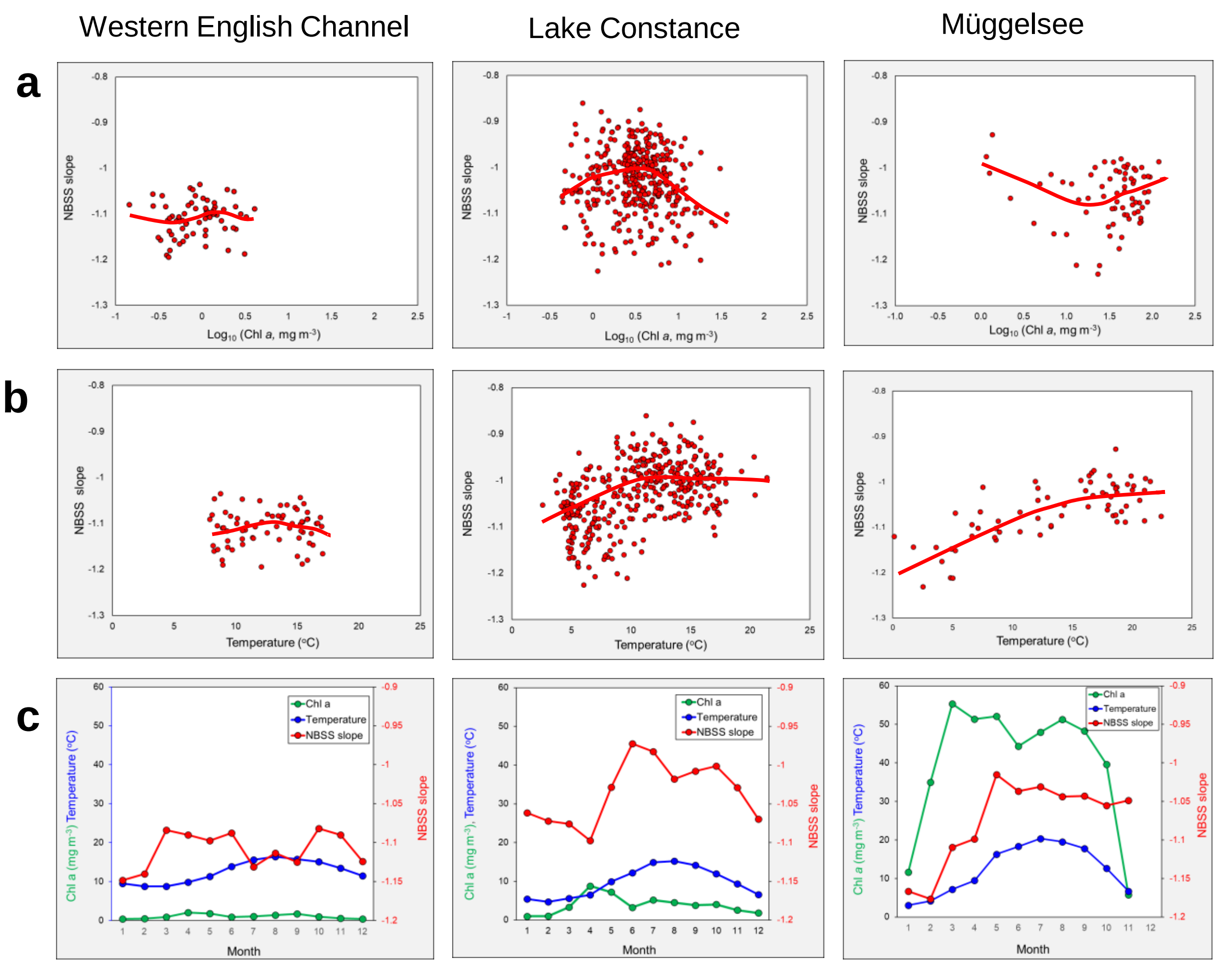


The station L4 (Western Channel Observatory[12], Lake Constance[17] and Müggelsee[18] all have high resolution, weekly time series data for the complete planktonic size spectrum (~10 orders of magnitude range in body carbon mass) available for multiple years (see **Supplementary Table 1** for more details). The ecosystems are compared here using the same scales to contrast the steeper NBSS slopes for L4 and the higher nutrient regimes in the two lakes (see **Fig. 2**). Panel **a** presents the NBSS slope against simultaneously-derived surface Chl *a* values for each determination (at each weekly time point for the two lakes and the weekly measurements averaged into months for L4 using data in ref-12). Panel **b** presents these NBSS determinations in relation to surface temperature. LOWESS lines in panels a and b suggest the underlying trends. Panel **c** plots average monthly time-course of Chl *a*, temperature and NBSS slope. The overall pattern is of high seasonality in all three systems, with substantial warming, spring and summer/autumn blooms and variable strengths of predator-prey coupling. The evolution of the cycles in NBSS slope are all partially out of phase with both bloom timing and the water temperature cycle, with the specific temporal dynamics of predator-prey interactions that cause

this described in more detail in the **Supplementary Discussion**. The overall picture emerging is that variable, system-specific dynamics drives a varied series of relationships between NBSS slope, Chl *a* and temperature. These include sometimes fundamentally different relationships between NBSS and Chl *a* and temperature to those in **Fig. 2**. These differences can be interpreted as travelling waves of biomass passing through the size spectrum following phytoplankton blooms that lever large variations in slope values, as described more fully by **Supplementary Fig 3** and ref-21.

**Supplementary Figure. 3. The shorter-term dynamics of size spectra is sensitive to the mass range over which they are measured, as well as to the system-specific seasonality.**

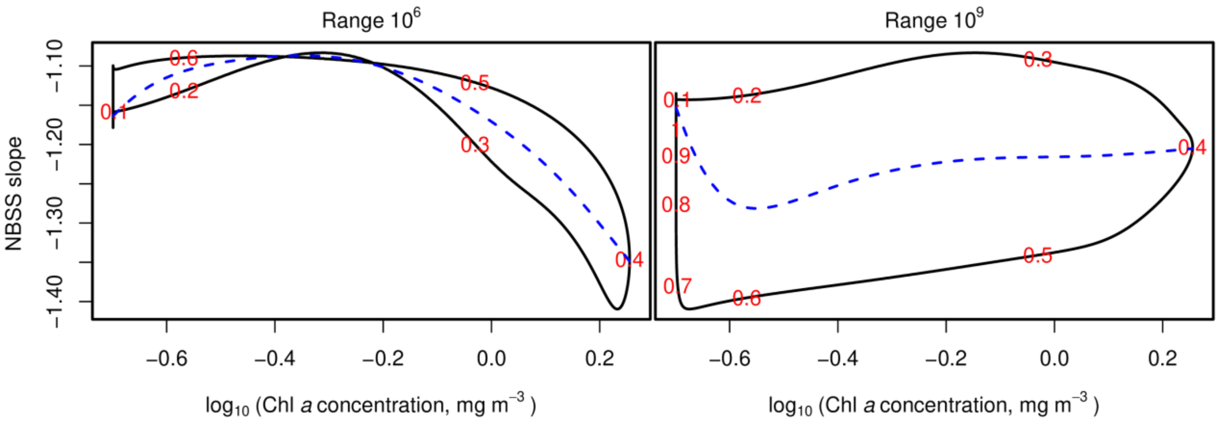


Comparison of snapshot slopes determined from the Non-linear Species Size-Spectrum Model[20,22] over two different body-mass ranges. We simulated a burst in Chl *a* concentration, which then triggered a "wave" travelling along the size-spectrum towards larger body masses with decreasing speed[22]. The Chl *a* pulse was modeled by adding to a baseline Chl *a* concentration of 0.2 mg $m^{-3}$ a time-dependent Gaussian that peaked 0.4 years after start of the simulation with a height of 1.6 mg $m^{-3}$ and a duration given by the Gaussian's `standard deviation' of 0.08 years. NBSS slopes were determined using linear regression of log-log plots over the indicated ranges (with the smallest size class at $0.8\times10^{-12}$ g C). In both panels, the black line traces simultaneous values of Chl *a* concentrations and NBSS slope sampled approximately every three days from simulations, with red numbers indicating the corresponding time in years. Simulated samples were then fitted using the gam function of the R package 'gam' with standard parameters (blue dashes). The Non-linear Species Size-Spectrum Model groups species according to maturation body mass into classes of equal width along the logarithmic maturation body mass axis. It simulates the changes through time in the biomass within each class that result from density-dependent growth, reproduction, and predation mortality. It then reconstructs the resulting dynamic NBSS, taking intraspecific population size structure into account. Model details and model parameters were as described in ref-20. Remarkably, the fits bend in opposite directions depending on the range over which the size spectrum is sampled.

**<u>Supplementary Fig. 4</u>. The projections of the supportable biomass of fish are more sensitive to the uncertainty in projected Chl *a* and phytoplankton biomass than to the uncertainty of our NBSS slope – Chl *a* relationship**

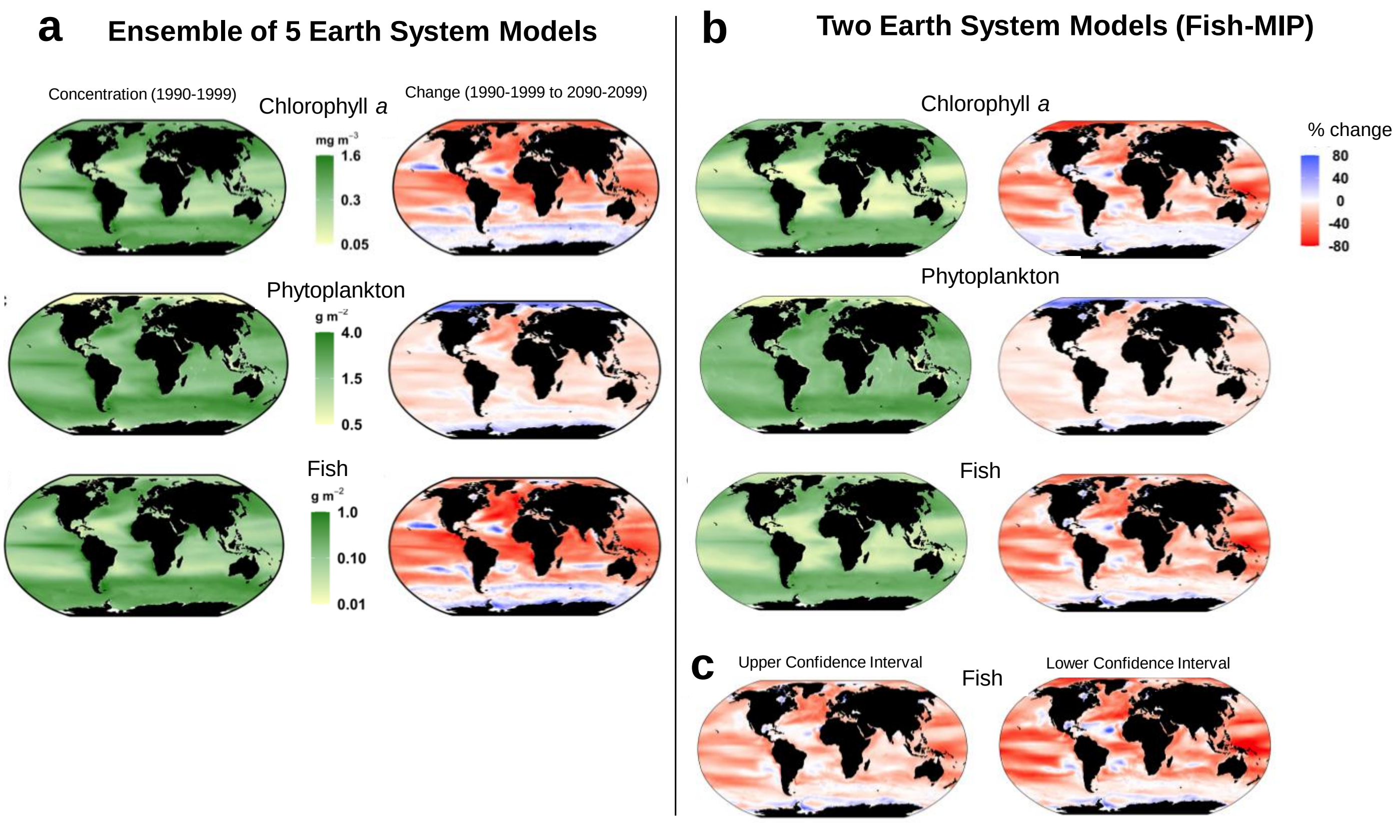


**a** Global maps[23] of surface Chl *a* concentrations, (mg Chl *a* $m^{-3}$), Phytoplankton Carbon (mg C $m^{-2}$) and Supportable biomass of fish 1990-1999 and percentage change from 1990-1999 to 2090-2099 from 5 ESMs (CESM2, GFDL-ESM4, IPSL-CM6A-LR, MPI-ESM1-2-HR, UKESM1-0-LL) from the Coupled Model Intercomparison Project Phase 6[24] under historical (1990-19990) and a single future (2015-2100) high emissions climate scenario (SSP5-8.5). **b** equivalent values based on just two of these models used for the Fish-MIP ecosystem model intercomparison[25], namely GFDL-ESM4 and IPSL-CM6A-LR and from the Coupled Model Intercomparison Project Phase 6[24] under historical (1990-2000) and a single future (2015-2100) high emissions climate scenario (SSP5-8.5). This panel b pertains to our study (main text Figs. 4 and 5), and shows a more homogonous picture of decline in phytoplankton biomass at low latitudes, and consequently a lower degree of contrast in the regional distribution of supportable fish biomass than when using a wider ensemble of 5 ESMs in panel a. For comparison, the respective global declines in phytoplankton and supportable fish biomass from panel a are 8.8% and 22%, whereas those from panel b are 7.5% and 19%. **c** changes in supportable biomass of fish, based on the two ESMs used in panel b, but driven by the equations in **Fig. 2a** describing

the upper and lower 95% confidence intervals for the NBSS slope – Chl *a* relationship. The similarity between these maps and the fish map in panel b, and their difference with that in panel a, illustrates that the main factor driving the estimates of supportable biomass of fish are the ESM projections of future Chl *a* and phytoplankton carbon, rather than the uncertainty over the NBSS-Chl *a* relationship.

Because our estimate of the supportable biomass of fish is driven both from modelled Chl *a* and phytoplankton carbon, the changes in modelled fish carbon biomass (for example in the Arctic) do not always relate simply to those of Chl *a*. In the Arctic, modelled surface Chl *a* declines whereas depth-integrated phytoplankton increases, and the two effects in combination lead to a general (albeit modest) projected decline in supportable fish biomass (**Fig. 4 g**). This result is in sharp contrast to model projections of a sharp increase in Arctic total consumer biomass[25] based on ensembles of food web models that include varying temperature-based parametrizations[26].

In summary, all of these projections of the supportable biomass of fish depend strongly on the earth system model output. However, discrepancies between food web model projections and those based on size spectra may provide useful insights for model inter-comparison and development.

# SUPPLEMENTARY DISCUSSION

## Seasonal dynamics of the Plymouth L4 station, Western English Channel

The Western English Channel dataset used here has 6 years of weekly resolution sampling[12], with size spectra then averaged into monthly blocks; 71 months being presented here. This site has counter-intuitive predator prey dynamics, whereby mesozooplankton consistently increase before the spring bloom. This likely reflects adequate late winter food, coupled to strong predation pressure later in the season[12]. March often sees an increase in meroplankton, helping to make this time of year have a relatively flat NBSS slope. Later in the summer, biomass of metazoan grazers continues to increase modestly, but there are substantial increases in pico- and nano-sized cells as well as dinoflagellates. This occurs despite the sustained low nutrient concentrations throughout summer, and continues to steepen the NBSS slope throughout the summer stratified period (typically May-September). Larger grazers are more prominent in autumn, but metazoans decline substantially towards late winter, leaving an assemblage strongly dominated by small cells and with a steep NBSS slope. Overall, these

counterintuitive predator prey dynamics produce steep NBSS slopes in both mid-summer and mid-winter and therefore little relationship between NBSS and temperature or Chl *a*.

**Seasonal dynamics of Lake Constance:**

Chl *a* increases from low winter values (complete mixing, no ice cover) when mixing reduces and nutrients are plentiful[17]. The peak is in spring when Chl *a*: biovolume and Chl: C ratios are high (i.e. fast growing, still partly light-limited algae). This peak then declines to the clear-water phase (CWP) with grazer-induced reduction of phytoplankton. There follows a recovery because herbivory declines due to food shortage and increasing carnivory by carnivorous zooplankton and fish (larvae). Chl *a,* however, does not rise so high as spring values, although summer algal biomass can surpass spring values. Towards autumn, Chl *a* declines with increasing vertical mixing and declining irradiance.

NBSS declines from January to April because the small phytoplankton increases together with ciliates whereas crustaceans cannot follow quickly at the still low temperature. The 2-3 largest size classes are almost empty because the large carnivorous crustaceans do not hibernate as plankton. In late May-June and July the herbivorous crustaceans such as daphnids reach their maximum and graze down the phytoplankton (CWP) causing the peak in the NBSS with the shallowest slopes. By around August phytoplankton has recovered and are more defended, i.e. moderate blooms of larger forms more resilient to predation such as pennate diatoms prevail, the herbivores comprise ciliates and crustaceans while the large carnivorous crustaceans peak at this time. In autumn another but less pronounced predator-prey cycle occurs as observed before and during the CWP. Later in autumn overall plankton biomass declines, particularly in crustaceans, with bacteria remaining relatively constant. As seen at Plymouth L4, this leads to relatively steep winter NBSS slopes.

**Seasonal dynamics of Müggelsee:**

The lake is shallow (mean depth 4.5 m), with a high range of winter to summer temperatures[18]. The lake is mostly polymictic, i.e. mixed more or less to the bottom except during short hot summer periods or during ice cover. The shallow depth means high irradiance and algal growth starts quickly without requiring stratification. There is an extended spring peak of rather productive algae per unit weight, then a brief CWP may occur in May. Cyanobacteria bloom strongly in summer, with low production to biomass ratio due to intensive self-shading. The blooms decline with low light, more intense mixing by wind and pronounced cooling by late autumn. These dynamics lead to a broadly similar cycle of NBSS as at Lake Constance, albeit without such a pronounced predator-prey cycle and overall higher ratios of producers to consumers. Also, the bacterial biomass is higher, extends to larger size classes and has less

seasonality. This implies relatively high biomasses year-round at the low end of the spectrum, reducing the slope.

## SUPPLEMENTARY REFERENCES


1. Rodriguez, J and M.M. Mullin. Relation between biomass and body weight of plankton in a steady state oceanic environment. *Limnol. Oceanogr*. **31,** 361-370. (1986).

2. Rochera, C., Queseda A., Torod, M., Ricoe, E. & Camacho A. Plankton assembly in an ultra-oligotrophic Antarctic lake over the summer transition from the ice-cover to ice-free period: a size spectra approach. *Polar Sci* **11**, 72-82 (2017).

3. Frangoulis, C. et al. Connecting export fluxes to plankton food-web efficiency in the Black Sea waters in-flowing into the Mediterranean Sea. *J. Plankton Res*. **32**, 1203–1216 (2010).

4. Frangoulis,C. et al. Expanding zooplankton standing stock estimation from meso- to metazooplankton: a case study in the N. Aegean Sea (Mediterranean Sea). *Cont. Shelf Res*. **149**, 151–161 (2017).

5. Quinones, R. A., Platt, T. & Rodriguez J. Patterns of biomass-size spectra from oligotrophic waters of the Northwest Atlantic. *Progr. Oceanogr.* 57, 405-427 (2003).

6. San Martin, E., et al. Variation in the transfer of energy in marine plankton along a productivity gradient in the Atlantic Ocean. *Limnol. Oceanogr*. **51,** 2084–2091 (2006).

7. Batziakas, S., Frangoulis, C., Tsiola, A., Nikolioudakis, N., Tsagaraki, T.M. and Somarakis, S. Hypoxia changes the shape of the biomass size spectrum of planktonic communities: a case study in the eastern Mediterranean (Elefsina Bay). *Journal of Plankton Research, **42*** 752-766. (2020).

8. Sprules, W. G., & Goyke, A. P. Size-based structure and production in the pelagia of Lakes Ontario and Michigan, *Can. J. Fish. Aquat. Sci*. **51**, 2603–2611 (1994).

9. Sprules, W. G. & Munawar, M. Plankton size spectra in relation to ecosystem productivity, size and perturbation. *Can. J. Fish. Aquat. Sci*. **43**, 1789-1794 (1986).

10. Yurista, P. M. et al. A new look at the Lake Superior biomass size spectrum *Can. J. Fish. Aquat. Sci*. **71,** 1324–1333 (2014).

11. Chapra, S. C. & Dolan, D. M. Great Lakes total phosphorus revisited: 2. Mass balance modeling. *J. Great Lakes Res*.**38**, 741–754 (2012).

12. Atkinson, A. et al. Increasing nutrient stress reduces the efficiency of energy transfer through planktonic size spectra. *Limnology and Oceanography* **66**, 422-437 (2021).

13. Sprules, W. G. Ecological change in Great Lakes communities—a matter of perspective. *Can. J. Fish. Aquat. Sci.* **65,** 1–9 (2008).

14. Tarling, G. A., Stowasser, G., Ward, P., Poulton, A.J., Zhou, M. & Venables, H. J. Seasonal trophic structure of the Scotia Sea pelagic ecosystem considered through biomass spectra and stable isotope analysis. *Deep-Sea Res. II*, **59-60**: 222-236 (2012).

15. Sprules, W. G., Casselman, J. M., & Shuter, B. J. Size distribution of pelagic particles in lakes. *Can. J. Fish. Aquat. Sci*. **40**: 1761-1769 (1983).

16. de Eyto, E. & Irvine, K. Assessing the status of shallow lakes using an additive model of biomass size spectra. *Aquat. Conserv*.**17**, 724–736 (2007).

17. Gaedke U. The size distribution of plankton biomass in a large lake and its seasonal variability. *Limnol. Oceanogr*. **37,** 1202-1220. (1992).

18. Gaedke, U., Seifried, A., & Adrian, R. Biomass Size Spectra and Plankton Diversity in a Shallow Eutrophic Lake. *Internat Rev Hyrdrobiol* **1**,1-20 (2004).

19. Tittel, J., Zippel, B., Geller, W. & Seeger, J. Relationships between plankton community structure and plankton size distribution in lakes of northern Germany. *Limnol. Oceanogr*. **43**, 1119–1132 (1998).

20. Rossberg, A. G., Gaedke, U. & Kratina, P. Dome patterns in pelagic size spectra reveal strong trophic cascades. *Nat. Commun*. **10**, 4396 (2019).

21. Gaedke, U. Identifying ecosystem properties: A case study using plankton biomass size distributions. *Ecol. Modelling* **63,** 277-298 (1992 b)

22. Rossberg, A.G. A complete analytic theory for structure and dynamics of populations and communities spanning wide ranges in body size. *Adv. Ecol. Res*. **46**, 429-522. (2012).

23. Posit team. RStudio: Integrated Development Environment for R. Posit Software, PBC, Boston, MA. URL http://www.posit.co/ (2023).

24. Eyring, V. et al. Overview of the Coupled Model Intercomparison Project Phase 6 (CMIP6) experimental design and organization. *Geosci. Model Dev*. **9**, 1937–1958 (2016).

25. Tittensor, D.P., et al. Next-generation ensemble projections reveal higher climate risks for marine ecosystems. *Nature Climate Change* **11**, 973–981 (2021).

26. Heneghan, R. F. et al. Disentangling diverse responses to climate change among global marine ecosystem models. *Progr. Oceanogr.* **198**, 102659 (2021).
